# Lactobacilli with probiotic potential in the prairie vole (*Microtus ochrogaster*)

**DOI:** 10.1186/s13099-015-0082-0

**Published:** 2015-12-30

**Authors:** Senait Assefa, Kathleen Ahles, Simone Bigelow, J. Thomas Curtis, Gerwald A. Köhler

**Affiliations:** Department of Biochemistry and Microbiology, Oklahoma State University Center for Health Sciences, 1111 West 17th Street, Tulsa, OK 74107 USA; Department of Pharmacology and Physiology, Oklahoma State University Center for Health Sciences, Tulsa, USA

**Keywords:** *Lactobacillus johnsonii*, Prairie vole, Probiotics, RAPD, Intestine, Mercury, Behavior

## Abstract

**Background:**

Recent research suggests integration of the intestinal microbiota in gut-brain communication which could lead to new approaches to treat neurological disorders. The highly social prairie voles are an excellent model system to study the effects of environmental factors on social behavior. For future studies on the role of probiotics in ameliorating disorders with social withdrawal symptoms, we report the characterization of intestinal *Lactobacillus* isolates with probiotic potential from voles.

**Methods and results:**

30 bacterial strains were isolated from the vole intestine and found to be distinct but closely related to *Lactobacillus johnsonii* using 16S rRNA gene sequencing and Random Amplification of Polymorphic DNA fingerprinting. *In vitro* characterizations including acid and bile tolerance, antimicrobial effects, antibiotic susceptibility, and adherence to intestinal epithelial cells were performed to assess the probiotic potential of selected strains. Since previous studies revealed that mercury ingestion triggers social deficits in voles, mercury resistance of the probiotic candidates was evaluated which could be an important factor in preventing/treating these behavioral changes.

**Conclusions:**

This study demonstrates that lactobacilli with probiotic potential are present in the vole intestine. The *Lactobacillus* isolates identified in this study will provide a basis for the investigation of probiotic effects in the vole behavioral model system.

**Electronic supplementary material:**

The online version of this article (doi:10.1186/s13099-015-0082-0) contains supplementary material, which is available to authorized users.

## Background

Interest in the use of probiotic bacteria to enhance intestinal health in humans and animals has been growing in recent years. Probiotics are “live microorganisms that, when administered in adequate amounts, confer a health benefit on the host” [1]. At the beginning of the 20^th^ century, the Ukrainian bacteriologist Elie Metchnikoff [2] suggested that health benefits were associated with the ingestion of lactic acid bacteria such a *Lactobacillus bulgaricus*. At present, many intestinal probiotics belong to the genus *Lactobacillus.* Lactobacilli are aerotolerant gram-positive bacteria that form an important portion of the normal human microbiotas of the oral cavity [3], gastrointestinal tract [3, 4], and female genitourinary tract [5–7]. Of the more than 150 [8] known species of lactobacilli, the “acidophilus complex” has received particular attention because of the reported probiotic properties of some members of this subgroup [9]. An example is the species *Lactobacillus johnsonii*. Several studies reported that *L. johnsonii* strains isolated from the human intestine undergo processes that are thought to be beneficial to human health, particularly in the areas of immunomodulation, pathogen inhibition, and cell attachment [10, 11]. In addition, accumulating clinical and scientific evidence highlights the important role of probiotic lactobacilli in the bidirectional communication of the gut-brain-axis [12–14]. Studies in mice on *L. rhamnosus* JB-1 treatment have shown alteration in the central gamma–aminobutyric acid (GABA) expression and modulation of emotional behavior and depression [13]. At present, however, the mechanisms how probiotics such as *L. johnsonii* could affect brain function are unclear, but proposed mechanisms involve, e.g., the bacterial production of neurotransmitter precursors or of chemical compounds that act as hormones or that stimulate vagal afferent pathways [13, 15, 16].

For the past two decades, prairie voles (*Microtus ochrogaster*) have been the dominant animal model in which to study the formation and maintenance of social affiliations [17, 18] and have been proposed as an important animal model in which to study disorders such as schizophrenia, autism, and the effects of traumatic brain injury, all of which negatively impact social functioning [19, 20]. Prairie vole social behavior has been well-characterized. Field studies show that prairie voles are highly social; pairs share a nest and parental duties and, in fact, both members of a pair often are found in the same trap [21]. In the laboratory, voles appear to avoid isolation by seeking out conspecifics, and in fact, voles suffer significant stress when isolated [22–26]. In contrast to more traditional laboratory animals, prairie vole social behaviors actually are remarkably similar to those of humans, even displaying characteristics such as long-term pair-bonding, care of offspring by both parents, and sharing of a nest even beyond the breeding season [27]. Further, autonomic responses in voles are more like those of humans than they are like those of other rodent species [28]. Importantly, both the behavioral repertoire and the physiology of voles are well documented (e.g., [27, 29–32], so there is a strong literature base upon which additional studies can rest. Given their social structure, prairie voles present an ideal animal model in which to study the of the role of the microbiota-gut-brain-behavior axis in mediating social affiliation and avoidance behaviors, mate choice, parental care and other complex social interactions.

The primary objective of this study was to lay the groundwork for probiotic studies in voles by isolating *Lactobacillus* strains with high probiotic potential from the vole intestine. Host adaption is an important factor for probiosis. Therefore, we chose to isolate vole strains rather than using probiotics originating from humans or other animals. Lactobacilli were isolated using enrichment media and subsequently classified by 16S rRNA gene sequencing which also allowed for PCR-based analyses of *Lactobacillus* abundance in the vole intestine. Since orally administered probiotics must survive passage through the highly acidic stomach and withstand the adverse intestinal environment, the strains’ acid tolerance and bile resistance were determined. Further characteristics such as antimicrobial activities against fungi and bacteria as well as adhesion to intestinal epithelial cell lines were examined. In addition we included an assessment of the strains’ resistance to mercury chloride. There is evidence that probiotic bacteria could bind many toxic compounds such as aflatoxin B1 [33], cyanotoxins [34], cadmium and lead [35–37] from environmental samples. In this study, the probiotic candidate strains’ resistance to mercury chloride was also determined because research by Curtis and coworkers [38] revealed social withdrawal symptoms specifically in male voles upon inorganic mercury ingestion. Resistant strains might be more likely to survive mercury exposure and exert beneficial effects on an exposed host organism. All lactobacilli isolated from the vole intestine in this study were closely related to *L. johnsonii* and several of the isolated strains exhibited potential for probiotic properties.

## Results

For purposes of characterizing the baseline state of vole gut lactobacilli, we have used same-sex cage mates. This eliminates the potential confounds of stress responses associated with social isolation or endocrine responses associated with reproductive activation, mating, and parental behavior [22–26]. Further research will be needed to assess whether and how the microbiota might change in pair-bonded and/or parental animals. Although these are important questions, they are beyond the scope of this paper, and will be addressed in subsequent studies.

### Isolation of *Lactobacillus* strains from the prairie vole intestine

Plating of intestinal content from prairie voles on *Lactobacillus* enrichment media resulted in the selection of 30 bacterial isolates for further analysis. Sequence analysis of the respective PCR amplicons generated with the well-conserved 16S rRNA gene primers 8F and 1491R revealed distinct but closely related matches (e.g. 98 % at 100 % coverage) with database entries of the 16S rDNA of *Lactobacillus johnsonii* (Table 1; Additional file 1: Figure S1). The 16S rDNA sequences of strains PV012, PV021, and PV034 also were confirmed by genome sequencing results (see Additional file 1: Figure S1).

**Table 1:** List of bacterial and fungal strains used in this study

Species	Strains	Origin	References
*Candida albicans*	SC5314	Clinical isolate	[73]
*Escherichia coli*	NovaBlue Singles	EMD millipore	http://www.emdmillipore.com
*Pseudomonas aeruginosa*	PA01	Clinical isolate	[74]
*Staphylococcus aureus*	ATCC 25923	Clinical isolate	http://www.atcc.org
*Lactobacillus* spp.	PV010–PV019	Vole cecum	This study
*Lactobacillus* spp.	PV020–PV021	Vole small intestine	This study
*Lactobacillus* spp.	PV022–PV023	Vole cecum	This study
*Lactobacillus* spp.	PV024–PV027	Vole colon	This study
*Lactobacillus* spp.	PV028–PV031	Vole small intestine	This study
*Lactobacillus* spp.	PV032–PV035	Vole cecum	This study
*Lactobacillus* spp.	PV036–PV039	Vole colon	This study
*Lactobacillus johnsonii*	ATCC 33200^a^	Human blood isolate	http://www.atcc.org
*Lactobacillus reuteri*	RC-14^a^	Human vaginal isolate	[75, 76]
*Lactobacillus rhamnosus*	LGG^a^	Human fecal isolate	[77]

*PV* prairie vole isolate

^a^Probiotic reference strain

### Strain differentiation by RAPD analysis

**Fig. 1:**
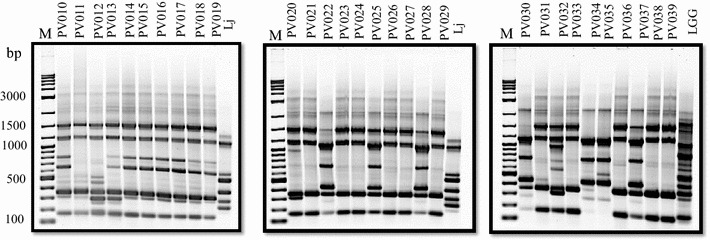
Random amplified polymorphic DNA (RAPD) analysis of 30 prairie vole *Lactobacillus* isolates. Amplified fragment patterns for RAPD primer 272 (see text) are shown after electrophoresis on 1.5 % agarose gels. PV010-PV039: Prairie vole *Lactobacillus* strains PV010-PV039; Lj: *L. johnsonii* ATCC 33200; LGG: *L. rhamnosus* GG. *M* New England BioLabs 2-log DNA ladder

**Fig. 2:**
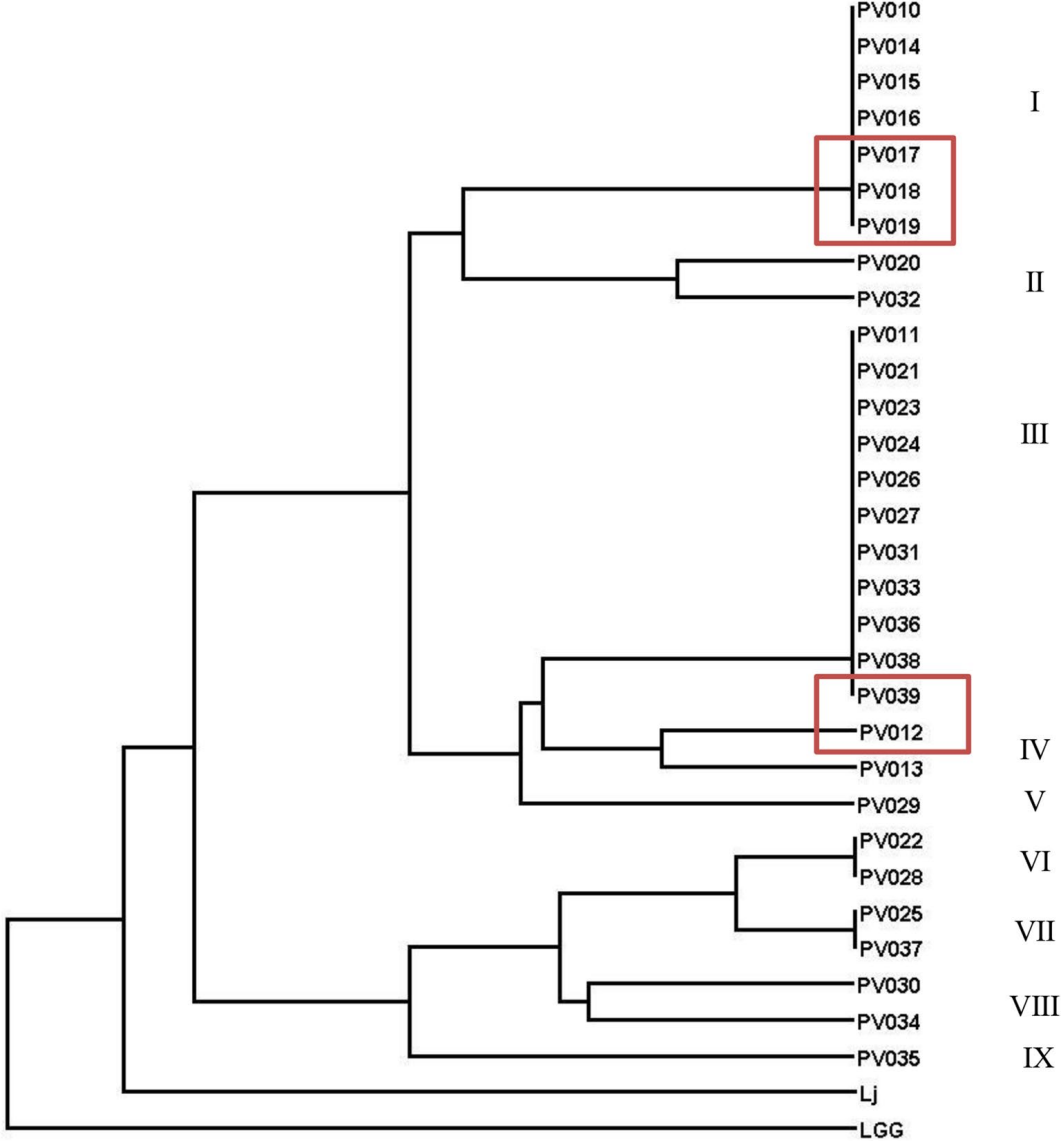
Random amplified polymorphic DNA (RAPD) analysis of 30 prairie vole *Lactobacillus* isolates. Dendrogram of the cluster analysis of RAPD results using the DendroUPGMA program (see “Methods”). Putative clustering is indicated with roman numerals. Strains with the overall best performance in this study are *boxed* in *red*

Due to their close relatedness, a RAPD typing technique was employed to genetically type the 30 prairie vole *Lactobacillus* isolates. To systematically examine the genetic fingerprints of the different strains, a set of three previously published RAPD primers (272, 277, and 287; [39]) was evaluated for differentiation of the bacterial strains. Primer 272 (see Table 2) was chosen for further analyses because it delivered the best discriminatory power by reproducibly amplifying five or more random DNA fragments ranging in size from approximately 180 bp to 3000 bp (Fig. 1). Twenty-seven of the 30 isolates share common bands at 175, 375, 1200 and 1500 bp (Fig. 1). In this regard, the RAPD fingerprinting was able to cluster genetically identical strains as well as differentiate distinct strains among the isolates. For instance, multiple strains such as PV010, PV014-PV019 or PV011, PV021, PV023, PV024, PV026, PV027, PV031, PV033, PV036, PV038 and PV039 were found to possess identical RAPD fingerprints suggesting that the isolates were identical or if genetic heterogeneity exists among these isolates, it could not be discriminated by RAPD. Overall, RAPD analysis of the 30 isolates revealed nine distinct clusters (Fig. 2). Notably, eight strains (PV012, PV013, PV020, PV029, PV030, PV032, PV034, and PV035) produced patterns with unique PCR bands (Fig. 2). RAPD bands at 1200, 650, 450, and 300 bp are shared with the human *L. johnsonii* ATCC 33200 strain by 23, 8, 11, and 12 isolates, respectively. In general, the RAPD fingerprinting analysis was effective for rapid differentiation within the different isolates. *L. rhamnosus* GG was included as reference strain and showed almost no RAPD pattern similarities to the vole intestinal strains.

**Table 2:** DNA oligonucleotide primers and hydrolysis probes used in this study

Primer	5′-Sequence-3′	References
8F	AGAGTTTGATCMTGGCTCAG	[65]
1491R	ACGGCTACCTTGTTACGACTT	[65]
RAPD 272	AGCGGGCCAA	[39]
1391R	GACGGGCGGTGTGTRCA	[78]
GK1053F	ATGGCTGTCGTCAGCTCGT	Adapted from [79]
GKUNI16STaqCCC	VIC-AACGAGCGCAACCC-MGB	This study
TaqLacF	TGGAAACAGATGCTAATACCG	[40, 41]
TaqLacR	CGTCCATTGTGGAAGATTCCCT	Adapted from [40, 41]
GKLPV16STaq	FAM-ACTGAGACACGGCCC-MGB	This study

### Abundance of lactobacilli in the prairie vole GI tract

**Fig. 3:**
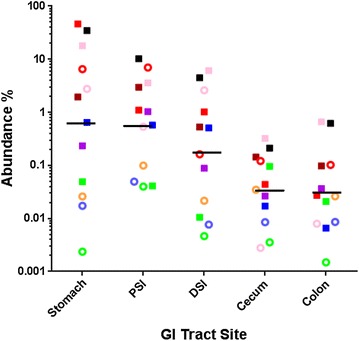
Relative abundance of lactobacilli in the GI tract of prairie voles. As indicator for the amount of lactobacilli in the vole GI tract, qPCR assays using group-specific and universal primers in conjunction with hydrolysis probes (see Table 2) were conducted to determine the relative abundance of *Lactobacillus* rRNA gene copies in content samples from the vole stomach, proximal small intestine (PSI), distal small intestine (DSI), cecum, and colon. Percent abundance values for five female (*ring symbols*) and seven male animals (*solid symbols*) are depicted on a logarithmic scale. Individual animals are represented by a specific *symbol-color* combination. Experiments were performed at least in duplicate. The *horizontal bars* indicate the geometric means of the abundance at the indicated sites for the twelve animals

We conducted a comparative survey to estimate the amount of lactobacilli present in male and female vole GI tracts by 16S rRNA-based qPCR. Published *Lactobacillus*-specific 16S rRNA gene primers were adapted to ensure complementarity with the respective gene sequences of the 30 vole strains, i.e., primer TaqLacR (Table 2) differs in one base from the published oligonucleotide sequence [40, 41]. Additionally, hydrolysis probes were designed for *Lactobacillus* and broad-range bacterial (primers GK1053F-1391R; Table 2) qPCR assays. These assays allowed for determination of the relative abundance of *Lactobacillus* 16S rDNA copy numbers in DNA isolated from vole stomachs, proximal and distal small intestines, ceca, and colons (Fig. 3). Interestingly, this assay revealed very high levels of lactobacilli in the stomachs (up to 47 %) and to lesser extend (up to 10 %) in the small intestines of some animals (see Fig. 3). Other animals exhibited far lower *Lactobacillus* abundance in the upper GI tract. In the distal GI tract (cecum and colon), lactobacilli appear to be generally less prevalent, accounting for less than 1 % of the total 16S rRNA. No statistically significant differences (ANOVA) were found in *Lactobacillus* abundance between the tested males and females at the respective gastrointestinal sites.

For purposes of characterizing the baseline state of vole gut microbiota, we have used same-sex cage mates. This eliminates the potential confounds of stress responses associated with social isolation or endocrine responses associated with reproductive activation, mating, and parental behavior [6–10]. Further research will be needed to assess whether and how the microbiota might change in pair-bonded and/or parental animals. Although these are important questions, they are beyond the scope of this paper, and will be addressed in subsequent studies.

### Acid tolerance of isolated *Lactobacillus* strains

**Fig. 4:**
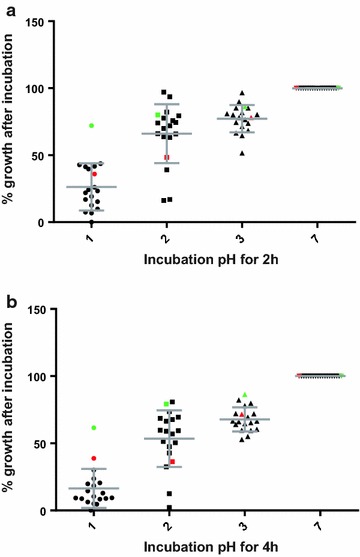
Acid tolerance of vole intestinal *Lactobacillus* isolates. Lactobacilli were incubated for 2 h (**a**) and 4 h (**b**) at various pH levels (pH 1, pH 2, pH 3, and pH 7) in PBS. Subsequently, the bacteria were inoculated in MRS and growth was determined after 24 h by OD_600nm_ measurement. Results are shown for the 17 most acid-tolerant *Lactobacillus* isolates as percent growth relative to growth after incubation at pH 7 (set to 100 %). *L. johnsonii* ATCC 33200 (*green data points*) and *L. rhamnosus* GG (*red data points*) were included as reference strains. While all strains tolerated prolonged incubation at pH 3 well, the depicted 17 strains survived pH 2 and some even pH 1. The reference strains appear to be more acid tolerant at pH 1 than the prairie vole strains. Data points are mean values from three experiments with duplicate measurements

The 30 vole intestinal *Lactobacillus* strains were screened for tolerance to strongly acidic conditions. Nearly all strains survived an incubation period of 4 h at pH 3, but only 17 strains were able to exhibit greater than 50 % growth at this pH level (Fig. 4). Data of strains which did not perform well are not shown. Figure 4 shows percent growth calculated from acid resistance assays at pH 1–3 during various incubation periods. The results indicated that the 17 selected strains survived during the 4 h incubation with some reduction in growth (20–44 %) compared to the control at pH 7. Although greater than 50 % of growth was suppressed, 12 of 17 strains survived pH 2 and pH 1 during the 4 h incubation. In general, little or no growth occurred in strains PV010, PV019, PV022, and PV037 following a 2 h incubation at pH 1. The results show that the acid tolerance of the investigated strains was variable, but comparable to the probiotic reference strains *L. johnsonii* ATCC 33200 and *L. rhamnosus* GG (Fig. 4). Overall, *L. johnsonii* ATCC 33200 appeared to be the most acid resistant strain.

### Resistance to bile and the bile acid taurocholate

Intestinal survival requires resistance to the antimicrobial components of bile. Therefore, the strains’ susceptibility to bile and the bile acid taurocholate was examined. Among the 30 isolated strains only 10 (PV011–PV014, PV017–PV019, PV021, PV024, and PV039) were resistant to high bile concentrations (0.5–8 %) within 24 h of exposure. Strain PV012 appeared to be the most resistant among these strains (Table 3) with an IC_50_ value of 4.2 % bile (comparable to human *L. johnsonii* ATCC 33200), whereas strain PV021 was the least resistant. The IC_50_ values for PV011, PV017–PV019 ranged between 2.7 and 3.6 %. The presence of 14 mmol/L taurocholate had no significant effect on the growth of eight strains (PV011–PV015, and PV017–PV019, see Table 3), but it significantly (*P* < 0.05) affected the growth rate of 14 strains (PV021–PV024, PV028–PV031, PV033, and PV034–PV039). Similar to the acid tolerance test, the bile and bile salt resistance levels of PV012 and PV017–PV019 were similar to the reference strains ATCC 33200 and LGG (Table 3).

**Table 3:** H_2_O_2_ production and IC_50_ values of human and vole *Lactobacillus* strains for bovine bile, taurocholate, and HgCl_2_

Strain	H_2_O_2_^a^	Bovine bile (%)^b^	Taurocholate (mmol/L)^b^	HgCl_2_ (mmol/L)^b^	
				24 h	48 h
PV010	−	<0.13	7 ± 0.3	0.1 ± 0.03	0.1 ± 0.00
PV011	−	3.0 ± 0.9	>14	0.1 ± 0.01	0.1 ± 0.01
PV012	−	4.2 ± 0.3	>14	0.2 ± 0.01	0.2 ± 0.04
PV013	−	1.6 ± 0.1	>14	0.1 ± 0.03	0.1 ± 0.03
PV014	−	1.8 ± 0.8	>14	0.1 ± 0.03	0.1 ± 0.03
PV015	−	0.8 ± 0.4	>14	0.1 ± 0.03	0.2 ± 0.23
PV016	−	<0.13	7 ± 1.2	0.2 ± 0.16	0.2 ± 0.23
PV017	−	2.8 ± 0.6	>14	0.2 ± 0.07	0.2 ± 0.10
PV018	−	3.3 ± 0.7	>14	0.2 ± 0.07	0.2 ± 0.11
PV019	−	3.6 ± 0.7	>14	0.1 ± 0.03	0.2 ± 0.13
PV020	−	<0.13	>14	0.2 ± 0.22	0.2 ± 0.22
PV021	−	0.4 ± 0.3	14	0.1 ± 0.10	0.2 ± 0.22
PV022	−	0.2 ± 0.1	9 ± 2.3	0.2 ± 0.06	0.2 ± 0.24
PV023	−	0.2 ± 0.1	10 ± 3.5	0.1 ± 0.12	0.2 ± 0.15
PV024	−	1.4 ± 0.5	14	0.1 ± 0.05	0.1 ± 0.09
PV025	+	<0.13	5 ± 1.2	0.1 ± 0.05	0.2 ± 0.23
PV026	−	<0.13	8 ± 1.2	0.2 ± 0.27	0.2 ± 0.25
PV027	−	<0.13	8 ± 1.7	0.1 ± 0.11	0.2 ± 0.12
PV028	−	<0.13	9 ± 0.3	0.1 ± 0.09	0.2 ± 0.23
PV029	−	<0.13	9 ± 1.7	0.2 ± 0.25	0.2 ± 0.25
PV030	+	0.2 ± 0.1	10 ± 2	0.2 ± 0.25	0.2 ± 0.26
PV031	−	<0.13	9 ± 0.7	0.1 ± 0.08	0.4 ± 0.23
PV032	−	0.13 ± 0.1	3 ± 1.6	0.3 ± 0.20	0.4 ± 0.12
PV033	−	0.2 ± 0.1	13 ± 1.2	0.2 ± 0.18	0.2 ± 0.10
PV034	+	0.2 ± 0.1	10 ± 1.2	0.1 ± 0.08	0.2 ± 0.11
PV035	−	0.3 ± 0.2	9 ± 1.0	0.3 ± 0.16	0.3 ± 0.12
PV036	−	<0.13	8 ± 3.9	0.1 ± 0.04	0.1 ± 0.05
PV037	+	0.2 ± 0.1	10 ± 2.3	0.1 ± 0.02	0.4 ± 0.07
PV038	−	<0.13	11 ± 2.6	0.2 ± 0.14	0.2 ± 0.11
PV039	−	0.8 ± 0.2	>14	0.1 ± 0.03	0.2 ± 0.05
Lj	+	4.0 ± 0.8	>14	0.02 ± 0.0	0.02 ± 0.0
LGG	−	1.0 ± 0.1	>14	0.1 ± 0.04	0.2 ± 0.11
RC14	+	n. d.	n. d.	n. d.	n. d.

^a^Purple color indicator for hydrogen peroxide production visible (+) or not visible (−) around colonies on ABTS agar

^b^IC50 values shown indicate the concentration of inhibitor (mean of triplicate experiments ± standard deviations) that led to 50 % growth reduction. For out of-out-range values the upper or lower concentration limits tested are shown. *L. johnsonii* ATCC 33200 (Lj), RC14, and LGG are included as reference strains

### Resistance to mercuric chloride

Lactobacilli have been suggested as candidate microorganisms that could aid in bioremediation and detoxification of heavy metals in the environment and in humans [37]. As a first step in the assessment of the capability of the investigated lactobacilli in mercury detoxification, we tested the strains’ resistance to different mercury chloride concentrations. The IC_50_ values are summarized in Table 3. Based on percentage growth at the initial 24 h incubation, most selected strains were found to be inhibited to 50 % of control growth by concentrations ≥0.1 mmol/L of HgCl_2_. In some strains (e.g., PV037), longer incubation to 48 h revealed adaptive effects, i.e. an increase in the IC_50_ value, suggesting the induction of resistance mechanisms. Overall, these results indicated that the tested vole strains and LGG tolerated similar HgCl_2_ concentrations in growth media while strain ATCC 33200 exhibited at least five-fold lower resistance (Table 3).

### Inhibition of pathogens

**Fig. 5:**
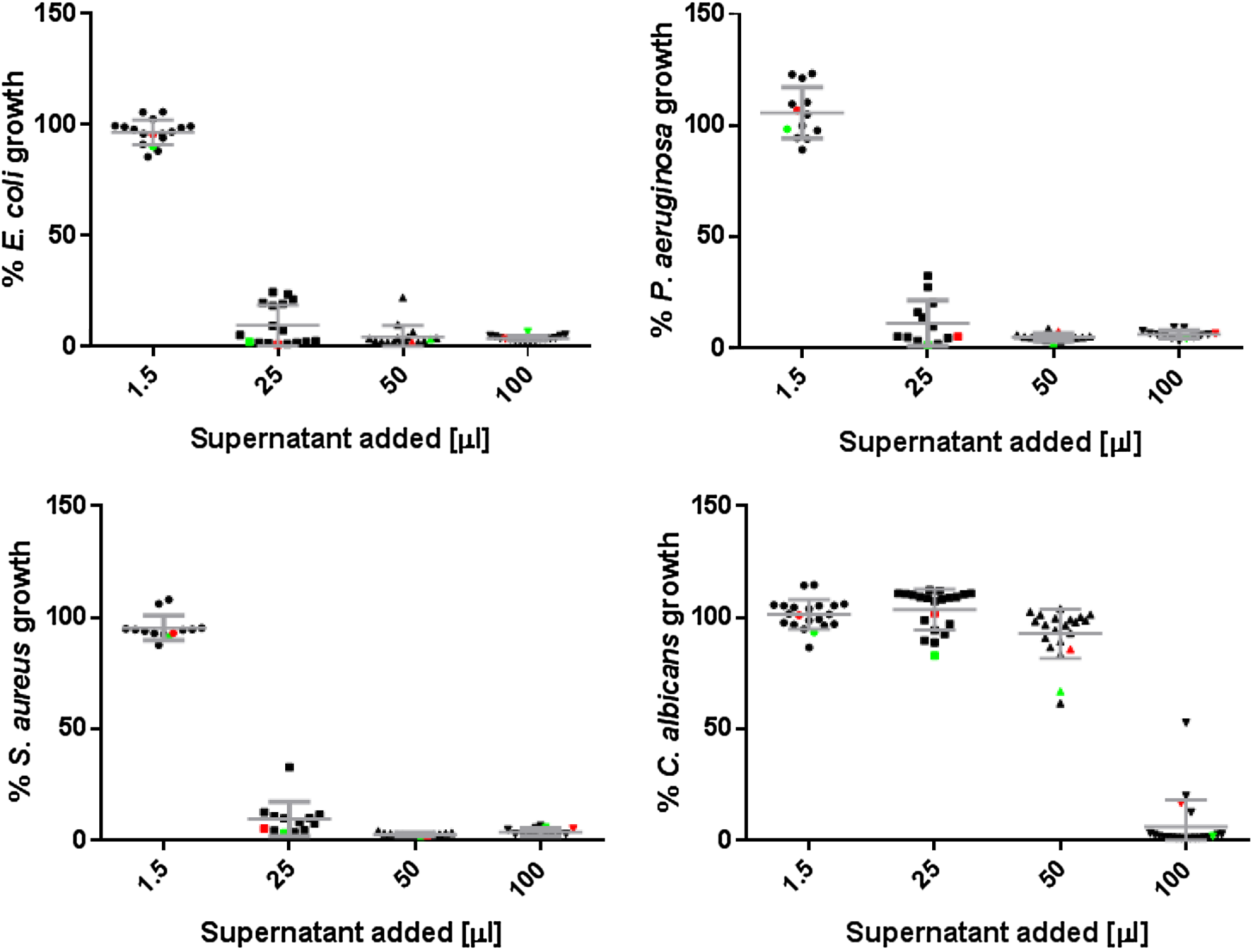
Antimicrobial effects of culture supernatants from vole *Lactobacillus* strains. Growth inhibition of *C. albicans*, *E. coli, P*. *aeruginosa, and S. aureus* in presence of the supernatants of the probiotic strains *L. rhamnosus* GG (LGG), *L. johnsonii* ATCC 33200 (Lj), and eleven selected strains of vole lactobacilli is depicted. Graphs depict the percent growth of the indicator microorganisms in 200 µL total culture volume following addition of 1.5, 25, 50 and 100 µL of *Lactobacillus* culture supernatants. Percent values were calculated from control growth, i.e., no supernatant added to the culture. Assay results are graphed for the most efficient strains in inhibiting bacterial growth. In general, antifungal activities towards *C. albicans* were less potent and only effective at high supernatant concentrations. Data points are mean values from three experiments with duplicate measurements. *L. johnsonii* ATCC 33200 (*green data points*) and *L. rhamnosus* GG (*red data points*) were included as reference strains

The antimicrobial activities of the vole *Lactobacillus* isolates were assessed by measuring the growth of the tester microorganisms *Candida albicans*, *Escherichia coli*, *Pseudomonas aeruginosa* and *Staphylococcus aureus* in the presence of the isolates’ culture supernatants (Fig. 5). Supernatants from 11 vole isolates (PV012, PV017–PV019, PV027, PV028, PV030, PV034, and PV037–PV039) and the two reference strains (ATCC 33200 and LGG) showed strong antagonistic activities towards all four tester microorganisms. The growth of the bacteria was inhibited at only 1/8th (25 in 200 µL total volume) dilution of these strains’ culture supernatants. Conversely, the growth of *C. albicans* was also inhibited by these strains, however, only at more elevated supernatant concentrations. In contrast to *C. albicans* and *S. aureus*, *E. coli,* and *P. aeruginosa* do not grow well in pure MRS broth. Therefore, we used LB broth to grow these bacteria and also tested whether addition of up to 50 % MRS would negatively influence growth. Compared to pure LB, growth of the bacteria was not significantly affected by addition of MRS broth alone (data not shown).

### H_2_O_2_ production

**Fig. 6:**
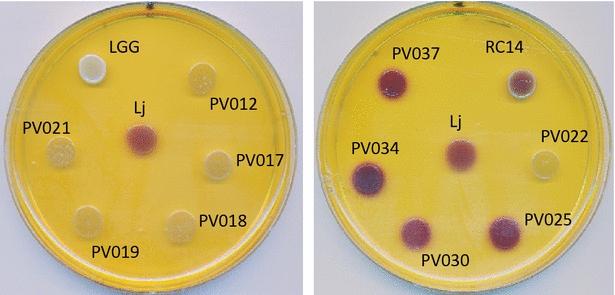
Hydrogen peroxide production by vole *Lactobacillus* strains. Representative ABTS agar assay plates indicating peroxide formation in the bacterial colonies are shown. All strains were evaluated following growth on ABTS/peroxidase indicator plates. In this example, colonies of PV025, PV030, PV034, and PV037 as well as the positive controls *L. reuteri* RC14 and *L. johnsonii* (Lj) produced H_2_O_2_ (*purple color*). *L. rhamnosus* GG (LGG) was included as negative control

The thirty vole *Lactobacillus* isolates and the reference strains ATCC 33200, LGG and RC-14 were evaluated for peroxide production on ABTS/peroxidase indicator plates. Among the 30 isolates, four were found to produce the potential antimicrobial factor H_2_O_2_ under the assay conditions. The colonies of PV025, PV030, PV034, and PV037 and ATCC 33200 as well as the reference strain RC-14 generated purple coloration on the plates indicating hydrogen peroxide production (+, Table 3; Fig. 6). Colonies from the remaining strains, including LGG, did not produce any detectable H_2_O_2_ in this assay (−). Anaerobic incubation of the four H_2_O_2_ producers precluded color formation.

### Biofilm formation

**Fig. 7:**
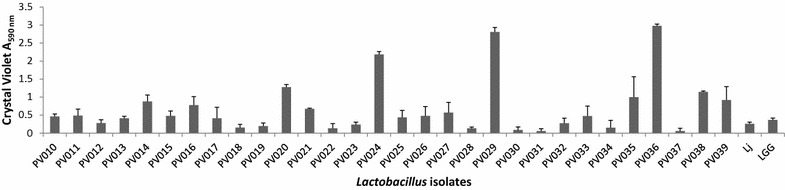
Biofilm formation on an abiotic surface. The *Lactobacillus* strains were incubated at 37 °C for 48 h in polystyrene culture dishes containing MRS medium. Biofilm formation was quantified using the crystal violet staining method. *Error bars* indicate standard deviations of three experiments with triplicate measurements

A crystal violet staining assay [42] was employed to test the 30 *Lactobacillus* strains for biofilm formation in tissue culture plates. Results are shown in Fig. 7. The assay revealed a wide range of variation in biofilm formation among the strains with statistically significant differences (*P* < 0.0001). Strain PV036 showed the highest biofilm production, whereas PV031 and PV037 were the lowest biofilm producers.

### Adhesion to the intestinal epithelial cell line Caco-2

**Fig. 8:**
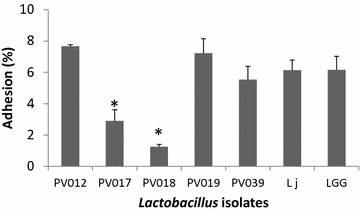
Adhesion of vole lactobacilli to Caco-2 epithelial cells. Assay results are depicted for the five most promising probiotic candidate strains as well as ATCC 33200 (Lj) and LGG as controls. Adhesion is expressed as the mean percentage of bacteria that bound to Caco-2 cell monolayers relative to the amount of bacteria added. The number of bacterial CFUs added varied between 1.5 × 10^8^ to 3.4 × 10^8^ CFUs mL^−1^. Each value represents the mean of triplicate measurements; *error bars* indicate the standard deviation.*P < 0.05 (one-way ANOVA)

The five most promising probiotic candidates were examined for adherence to intestinal epithelial cells. Three strains showed strong adherence levels to human Caco-2 intestinal epithelial cells, similar to the adherence observed with the human intestinal probiotic *L.* *rhamnosus* GG and *L. johnsonii* ATCC 33200 (Fig. 8). Strain PV012 was the most adherent strain in the assay since approximately 7.7 ± 0.1 % of the added bacteria bound to Caco-2 cells. PV018 was the least adherent (1.3 ± 0.2 %). The adhesion of PV018 and PV017 was significantly (*P* < 0.05) lower than the adhesion of PV012, PV019, PV039, ATCC 33200, and LGG.

### Antibiotic susceptibility

The susceptibilities of the five probiotic candidates to eight antibiotics from different antibiotic classes were determined by broth microdilution testing. Relatively low MICs were found with clindamycin, erythromycin, ampicillin, and doxycyclin (see Additional file 2: Table S1). However, the strains and controls (ATCC 33200 and LGG) were highly resistant to the aminoglycoside antibiotic neomycin.

## Discussion

It has become evident that the gut microbiota can influence host physiology, gut brain-communication, brain function and behavior [16]. The emerging concept of intricate involvement of the gut microbiota in the bidirectional communication between the enteric and central nervous systems (gut-brain-axis) raises the possibility of modulation of the integrated neuronal, hormonal and immune pathways by administration of probiotics [13]. In the present study, we isolated and characterized thirty *Lactobacillus* strains closely related to *L. johnsonii* from prairie voles, animals which have been suggested as a powerful experimental model in which to study the social brain [43]. In light of the in vitro results reported here, we propose the selection of five strains with high probiotic potential for further studies on the potential role of probiotics in modulating neurological disorders that are associated with social withdrawal symptoms.

Lactobacilli are commonly associated with the gastrointestinal tract of animals and humans, as also evidenced by our *Lactobacillus* abundance results in the vole GI tract (see Fig. 3). Interestingly, these results suggest high numbers of lactobacilli in the stomachs of some animals. Voles, like many herbivorous small mammals, are known to be coprophagic [44]. However, to what extent coprophagy serves to maintain a steady supply of microorganisms, in addition to the well-established nutritional benefits of coprophagy [44], is unknown. Thus, it is unclear whether the relatively high concentrations of lactobacilli in the stomach are the result of recent ingestion of fecal material, or are representative of the normal stomach microbiota in voles.

Probiotic effects of lactobacilli are based on adaptation factors for survival in the host’s gastrointestinal tract and probiotic factors for competition with pathogens and further health-promoting interactions with the host [45]. *L. johnsonii* appears to be the main species of lactobacilli inhabiting the human gastrointestinal tract and some *L. johnsonii* strains have been shown to exert probiotic effects [11, 46, 47]. Factors characteristic of *L. johnsonii* probiotics encompass immunomodulation, the ability to adhere to mammalian cells, and pathogen inhibition through production of antimicrobial substances such as lactic acid and bacteriocins. As an example, *L. johnsonii* NCC533 exerts antimicrobial mechanisms against several pathogens in vitro, including pH reduction, and lactic acid, bacteriocin and H_2_O_2_ production [48–50]. Here, we report that even though the isolated strains share highly similar 16S rRNA gene sequences to the species *L. johnsonii*, not all *Lactobacillus* isolates in this study produced the antimicrobial H_2_O_2_ under the test conditions. Nonetheless, the four H_2_O_2_ producers were among the eleven isolates exerting potent antibacterial and even antifungal effects. Although the identities of the inhibitory substances generated by the vole isolates have not been characterized, the broad inhibitory effects against the indicator bacteria and fungi are likely to be due to production of peroxide, organic acids such as lactic acid, bacteriocins, and other antimicrobial substances as reported for many probiotic *Lactobacillus* strains [45, 51]. Lactic acid production in concert with a low pH microenvironment leads to increased cellular toxicity due to diffusion of the undissociated acid into cells and subsequent intracellular acidification which could also promote synergism with other antimicrobial components [45, 52, 53]. Alakomi and coworkers [54] stated that lactic acid, in addition to its antimicrobial property based on lowering of the pH, also functions as a permeabilizer of the outer membrane of gram-negative bacteria, and thus potentiates susceptibility to other antimicrobial molecules.

During passage through the stomach, orally administered probiotics are exposed to high levels of acid stress. The pH value of gastric juice can vary in the range from 1.5 to 4.5 in a 2 h period [55]. Thus, probiotic candidates destined to benefit intestinal function must be able to remain viable after several hours in a highly acidic environment. As demonstrated, 17 of the investigated strains revealed acid tolerance after 4 h incubation under strong acidic conditions and were able to retain cell viability (Fig. 4). The results are similar to previous reports [56, 57] which suggests that the isolated strains have the ability to passage through the stomach without sustaining severe damage.

Resistance to bile and bile acid is another important adaptive factor of probiotics in the intestinal tract. Reports regarding the composition of bile juice from different animals are limited; as a result, most studies used ox gall (bovine bile) as a substitute. The average bile concentration is around 0.3 % and may range up to 2 % during the first hour of digestion [58]. We used bovine bile at concentrations ranging from 0.13 to 8 % to assess bile tolerance of the *Lactobacillus* strains. Previous reports stated that lactobacilli tolerated on average 0.3 % [51, 55, 56]. After 24 h incubation, five strains including ATCC 33200 exhibited IC_50_ values >2 % while the probiotic reference strain *L. rhamnosus* GG showed an IC_50_ of 1 %. Exposure to bile is accompanied by mild acid stress. Therefore, bile resistance is based on hydrolysis of bile (salts) and mechanisms of acid tolerance [45]. Future studies will reveal why some of the isolated strains can withstand such high bile concentrations.

We also investigated the strains’ susceptibility to inorganic mercury, a trait usually not considered in the characterization of probiotics. However, intestinal bacteria, including lactobacilli, play important roles in intestinal homeostasis, and their susceptibility to toxic metals could be of importance in certain gastrointestinal and/or neurological diseases induced by these metals [59]. Administering probiotics resistant to toxic metals could be an important factor to ameliorate metals-induced neurological disorders, including those associated with social withdrawal symptoms. A goal of this study is the identification of strains with high resistance to mercury chloride in combination with potent probiotic properties, which could potentially be used in future prophylactic or therapeutic interventions in the prairie vole animal model of mercury effects on social behavior [38]. Our in vitro studies revealed that some vole *Lactobacillus* isolates, in contrast to the lower tolerance of ATCC 33200, had IC_50_ values as high as 125 μmol/L which is 16,968 times the recommended maximum level of inorganic mercury for human consumption (2 ppb, [60]). A few strains (e.g., PV012, PV037; see Table 3) showed adaptation when the 24–48 h exposures to HgCl_2_ were compared. Higher IC_50_ values at 48 h could be due to induction of resistance genes/mechanisms, e.g., exerted by detoxifying proteins such as mercuric reductase (*merA*) or metal transporters. Interestingly, an unpublished draft genome sequence of strain PV012 generated via Ion Torrent PGM sequencing by our laboratory revealed the presence of a potential *merA* gene (data not shown). This gene might be expressed during extended inorganic mercury exposure to convey detoxification processes. Additionally, binding and sequestration of toxic metals by lactobacilli could be a possible remedial process which could be another probiotic health effect [37, 61, 62].

Probiotic bacteria can generate biofilms in the intestinal tract, albeit isolated cells and microcolonies appear to be more frequently encountered forms of colonization [45]. Nevertheless, we evaluated the strains for biofilm formation on an abiotic surface (polystyrene) in standard MRS medium without biofilm-promoting stressors (e.g., bile addition or omission of glucose; [63, 64]. Strains PV024, PV029, and PV036 showed the highest biofilm production (Fig. 7), however, these strains exhibited low degrees of antimicrobial activity and tolerance to bile and/or acidic pH conditions. Thus, under our assay conditions the ability to form biofilms on abiotic surfaces was negatively correlated with probiotic potential.

Adhesion of probiotic bacteria to the intestinal mucosa is considered another important adaptation factor for probiotic activity [45]. Several components of the bacterial cell surface appear to participate in the adherence of the bacterial strains to intestinal epithelial cells. Adhesion properties are strain characteristics and cannot be generalized to the species and therefore have to be individually tested [45]. In this study, we identified five strains that combined high resistance to acid, bile, and metal toxicity with potent antimicrobial properties and assayed their adhesion to the human intestinal cell line Caco-2. PV012 was the most adhesive strain followed by PV019 and PV039. The observed adhesion percentages were comparable with previous studies [11, 57]. Moreover, the adherence of both PV012 and PV019 was comparable or even better than that of ATCC 33200 and LGG (see Fig. 8). At present little is known whether adhesion of the vole isolates is regulated by inter- or intra-species signaling (quorum sensing) or which cell envelope components are involved in the adhesion process. However, strong adhesion to cells from a non-adapted host suggests a more generalized adhesion mechanism. Future studies will help to elucidate whether adhesion properties are correlated with probiotic effects in vivo.

The antibiotic susceptibility profiles of the prairie vole *Lactobacillus* strains (see Additional file 2: Table S1) could be of interest for future genetic manipulations of these bacteria and also for studies on the effects of antibiotics on the vole gastrointestinal microbiome.

Most importantly, future studies will investigate whether the isolated *Lactobacillus* strains are capable of influencing brain function and thereby altering behavior. In this context, probiotic effects on the highly developed social behavior of these animals will be of particular interest.

## Conclusions

Through the combined use of enrichment media, 16S rRNA gene sequencing and molecular strain typing, we isolated and differentiated thirty *Lactobacillus* strains from the prairie vole intestine. The described characterization of a set of adaptive and probiotic factors led to the selection of five vole *Lactobacillus* strains: PV012, PV017, PV018, PV019, and PV039. The selected strains showed evidence of potent antibacterial and antifungal properties, strong adherence to intestinal epithelial cells as well as resistance to bile and low pH. Moreover, they could potentially be employed in intestinal detoxification of inorganic mercury. Thus, the selected strains meet important prerequisites to study probiotic health effects in the prairie vole social behavior model.

## Methods

### Strains and culture conditions

Bacteria and fungi used in this study are shown in Table 1. Bacterial cultures were routinely grown in Difco Lactobacilli MRS broth (de Mann, Rogosa and Sharpe medium for lactobacilli; BD Diagnostics, Franklin Lakes, NJ, USA) or Luria–Bertani broth (LB Miller, Fisher Scientific, Pittsburgh, PA, USA; for *E. coli*, *Staphylococcus aureus,* and *Pseudomonas aeruginosa*) at 37 °C. YPD medium (Fisher Scientific; 10 g/L yeast extract, 20 g/L tryptone, 20 g/L dextrose) was used for growing *Candida albicans*. Solid media were generated by adding 15 g/L (bacteria) or 20 g/L (fungi) agar to the respective media. Stock cultures were maintained at −80 °C with 15 % v/v glycerol as cryopreservative.

### Animal care and handling

The voles used in this study were sexually-naïve adult (>60 days of age) male and female prairie voles (*Microtus ochrogaster*) from a laboratory breeding-colony descended from an Illinois population and were of the F4 and F5 generations relative to most recent out-crossing with wild stock. Voles are housed at 21 °C with a 14:10 light:dark cycle. Breeding pairs are housed in plastic cages (20 × 25 × 45 cm) containing corncob bedding with hay as nesting material. Ad libitum food (Purina rabbit chow supplemented with black-oil sunflower seeds) and water are available. After weaning at 21 days of age, offspring are housed in same-sex pairs in plastic cages (10 × 17 × 28 cm) until used in experiments. Except for the breeding pairs, sexes are maintained in separate rooms until used in experiments. The general experimental manipulations and animal handling procedures were approved by the Oklahoma State University Center for Health Sciences Institutional Animal Care and Use Committee.

### Bacterial strain isolation from the prairie vole GI tract

The bacterial strains isolated and characterized in this study are shown in Table 1. Two animals from each sex were euthanized and duplicate intestinal specimens were collected from the cecum, small intestine and colon. Following suspension of intestinal content in 0.5 mL sterile water, a dilution series (10^0^–10^−5^) was prepared for each sample and 100 μL of each dilution were cultured immediately on MRS agar plates. Samples from different animals or sites were kept separate. Enrichment for lactobacilli was achieved under anaerobic growth conditions at 37 °C for 48 h using a GasPak™ 100 container and EZ Anaerobe Pouch system (BD Diagnostics). Subsequently, bacterial colonies were randomly selected (up to 10 colonies per plate) and sub-cultured at least twice for purification. Only isolates with good and uniform growth on MRS agar were considered for further study. Following repeated purification, a distinct colony from selected isolates was used as inoculum for liquid MRS cultures. After 24–48 h of growth, frozen stock cultures with 15 % (v/v) glycerol as cryopreservative were prepared from these cultures. Working cultures were routinely propagated from the stocks aerobically or anaerobically.

### *Lactobacillus* DNA extraction, PCR and 16S rRNA-based identification

DNA extractions were performed from each of the thirty isolates. Bacterial DNA was isolated from 10 ml MRS broth culture grown overnight using a ZR Fungal/Bacterial DNA MiniPrep™ kit (Zymo Research, Irvine, CA, USA) following the manufacturer’s instructions. In brief, bacterial cells were harvested by centrifugation at 4500×*g* for 10 min at 4 °C and re-suspended in 750 µL of lysis buffer and added to ZR Bashing Bead Lysis tubes. A Mini-Beadbeater-96 (Biospec Products, Bartlesville, OK, USA) was employed for cell disruption. The resulting crude bacterial cell homogenates were processed for genomic DNA isolation according to the kit’s instructions. DNA concentrations were determined using a BioTek Synergy 2 Multimode Microplate Reader (BioTek Instruments, Inc. Winooski, Vermont).

The universal primers 8F and 1491R (see Table 2) were used to generate PCR amplicons of the bacterial 16S rRNA genes [65]. PCRs were carried out in a PTC-200 DNA Engine thermocycler (Bio Rad, Hercules, CA, USA) in 50 µL reactions employing AmpliTaq Gold 360 Master Mix (25 µL, Life Technologies, Carlsbad, CA, USA), 0.2 μM of 8F/1491R primer mix, and 1–2 µL bacterial DNA solution (100 ng) following the manufacturer’s guidelines. Amplification parameters consisted of an initial denaturation step at 95 °C for 10 min followed by 30 cycles of 15 s at 95 °C, 30 s at 55 °C, and 90 s at 72 °C. A final extension step at 72 °C for 10 min completed the reactions. Aliquots of the PCRs were evaluated by gel electrophoresis on 1 % agarose gels. Successful PCRs were purified and concentrated using the ZR DNA Clean and Concentrator 25 kit (Zymo Research) according to the manufacturer’s instructions.

Sanger sequencing of the isolates’ 16S rRNA gene amplicons was performed for species identification. PCR amplicons from twelve isolates were cloned in the pCR4-TOPO vector (TOPO TA Cloning Kit, Invitrogen, Carlsbad, CA, USA) for sequencing, whereas the remaining eighteen PCR amplicons were directly sequenced. The latter approach yielded results faster, while still providing the sequence information necessary for classification of the strains. Recombinant plasmids were transformed into *E. coli* Novablue Singles™ competent cells (EMD Millipore, Billerica, MA, USA) by electroporation using an ECM 399 electroporation system (BTX Harvard Apparatus, Holliston, MA, USA). Plasmids from successful transformations were isolated with the Zyppy Plasmid Midiprep kit (Zymo Research) following the manufacturer’s instructions and then sequenced. Amplicons/plasmid inserts were sequenced from both directions at the OSU Stillwater Recombinant DNA/Protein Core Facility. For classification of the isolates, the assembled sequences were compared to published 16S rDNA sequences in the NCBI GeneBank and Greengenes databases (http://www.greengenes.lbl.gov/blast; [66]) using the BLAST tool.

### Determination of the relative abundance of lactobacilli in the prairie vole GI tract

The relative abundance of *Lactobacillus* 16S rRNA gene copies in various regions of the prairie vole gastrointestinal tract (stomach, proximal and distal small intestine, cecum, and colon) was determined by exonuclease-based quantitative real-time PCR (qPCR). For this purpose, DNA was isolated from gastrointestinal contents of five female and seven male animals (one sample per site) using the ZR Fecal DNA MiniPrep kit (Zymo Research) according to the manufacturer’s directions. A group-specific assay to detect lactobacilli was designed employing the primer pair TaqLacF-TaqLacR in conjunction with the hydrolysis probe GKLPV16STaq (Table 2). For normalization across samples, qPCR assays with broad-range primers (GK1053F-1391R) and the hydrolysis probe GKUNI16STaqCCC were used. Quantitative PCR reactions were run on Applied Biosystems StepOne™ or 7500 real-time PCR systems using the TaqMan Universal Master MixII with UNG reagents (Life Technologies) and the following reaction parameters: UNG incubation 2 min at 50 °C, polymerase activation 10 min at 95 °C, 40 cycles of denaturation (30 s at 95 °C), annealing (30 s at 52 °C), and extension (90 s at 65 °C). Ribosomal RNA copy numbers were determined by comparison of quantification cycle values (Cq) of sample assays with standard curves generated with pLBB4c, a plasmid containing a *Lactobacillus* 8F-1491R 16S rRNA gene fragment that provided a quantified template for both targets. Assays were replicated at least in duplicate. Relative abundances of *Lactobacillus* 16S rRNA gene copies in each sample were calculated as percentages of the respective broad-range PCR values.

### Random amplified polymorphic DNA (RAPD) fingerprinting

Randomly amplified polymorphic DNA analysis was used to genetically differentiate the isolated *Lactobacillus* strains. For RAPD fingerprinting, the same bacterial DNA extracts were used as for cloning and sequencing so that the results could be directly matched. RAPD analysis was adapted from a previously described procedure [39]. The oligonucleotide primer RAPD 272 (see Table 2) was used throughout the study. PCRs were run on a PTC-200 DNA Engine thermocycler (Bio Rad) in 25 µL reactions employing AmpliTaqGold 360 Master Mix (12.5 µL, Life Technologies, Carlsbad, CA, USA), 10 µmol/L primer and 100 ng template DNA. PCR cycles were performed as follows: (1) 4 cycles of 94 °C for 5 min, 36 °C for 5 min (70 s ramp time), and 72 °C for 5 min (70 s ramp time), (2) 30 cycles of 94 °C for 1 min (55 s to heat from 72 °C), 36 °C for 1 min. (60 s ramp time), 72 °C for 2 min (70 s ramp time); and (3) a final extension of 72 °C for 6 min followed by a hold at 4 °C. All *Lactobacillus* strains were processed in duplicate to ensure RAPD typing was reproducible and reliable. The probiotic strains *L. johnsonii* ATCC 33200 and *L. rhamnosus* GG were used as references. PCR amplicons were separated by gel electrophoresis using 1.5 % high resolution agarose gels in 1× Tris–Acetate-EDTA buffer with a 100 bp DNA ladder (New England Biolabs, Ipswich, MA, USA) as size marker. Gels were stained with SYBR Safe DNA gel stain (Life Technologies) and scanned with a Typhoon 9410 Variable Mode Imager (GE Healthcare Biosciences, Pittsburgh, PA, USA). The resulting fingerprint bands were analyzed with Image Quant TL software (GE Healthcare) and PCR fragments patterns for each strain were determined. These amplicon patterns were used for cluster analyses to compare RAPD results of the *Lactobacillus* strains using “DendroUPGMA” (http://genomes.urv.cat/UPGMA/; [67]).

### Acid tolerance test

Freshly grown *Lactobacillus* cultures were pelleted at 4500×*g* for 10 min at 4 °C, washed twice and re-suspended in sterile phosphate-buffered saline (PBS, pH 7.2). Each pellet was diluted to OD_600nm_ = 0.05 in PBS at pH 1–5, 7 (adjusted with 1.0 N HCl) and incubated at 37 °C for 1, 2, and 4 h. Subsequently, 20 µL of the bacteria were inoculated into Cellstar 96-well tissue culture plates (Greiner Bio-One, Monroe, NC, USA) containing 180 µL of MRS broth and incubated at 37 °C for 24 h. Growth was determined by OD_600nm_ readings on a microplate reader.

### Bile and bile acid tolerance test

The method described by Ehrmann and coworkers [56] was used for testing tolerance to bovine bile (B3833, Sigma-Aldrich) and taurocholate (T4009, Sigma-Aldrich). Following overnight cultures in MRS, the bacteria were inoculated to a starting OD_600nm_ = 0.05 in 96-well tissue culture plates containing MRS with dilution series of bovine bile (0.13, 0.25, 0.5, 1, 2, 4, and 8 % w/v) or taurocholate (0.2, 0.4, 0.9, 1.8, 3.5, 7.0, and 14 mmol/L). MRS broth without addition of inhibitors was used as control. After incubation for 24 h, growth was determined by OD_600nm_ readings. Each assay was carried out in duplicate wells and repeated three times.

### Antimicrobial effects towards bacteria and fungi

The antimicrobial activity in *Lactobacillus* culture supernatants was tested as previously described by Lee and coworkers [68]. Briefly, the vole *Lactobacillus* strains and the two reference strains (ATCC 33200 and LGG) were cultured overnight in 6-well tissue culture plates (Cellstar, Greiner Bio-One). Cell-free supernatants were harvested by centrifugation for 5 min at 4000×*g*, sterilized with 0.2 µm polyethersulfone membrane syringe filters, and 100, 50, 25, 12.5, 6.25, 3.12, and 1.6 µL of the supernatants were pipetted into Cellstar 96-well tissue culture plates. The volume in each well was adjusted to 100 µL with MRS broth. As indicator microorganisms, the pathogens *P. aeruginosa*, *S. aureus*, *C. albicans*, and the non-pathogenic K-12 *E. coli* strain NovaBlue Singles (see Table 1) were added in 100 µL fresh medium (LB for *E. coli* and *P. aeruginosa*, MRS for *C. albicans* and *S. aureus*; all adjusted OD_600nm_ = 0.01) into the wells containing *Lactobacillus* supernatants and incubated for 24 h at 37 °C. Pure cultures of each indicator microorganism were included as controls. Growth of the indicators was assessed by optical density readings at 600 nm in a Biotek Synergy 2 microplate reader. The assay was carried out in duplicate wells and repeated three times with all microbial cultures prepared fresh from frozen stocks.

### Analysis of H_2_O_2_ production

The ability of the isolates to produce hydrogen peroxide was determined qualitatively using the 2,2′-azino-bis(3-ethylbenzoline-6-sulfonic acid) diammonium salt (ABTS)-MRS agar method [69]. Thirty mg of ABTS (Sigma, St. Louis, MO, USA) and 2 mg of horseradish peroxidase (Sigma, St. Louis, MO, USA) were dissolved in 10 mL distilled water and filter-sterilized. This ABTS/peroxidase solution was added to 90 mL of MRS agar that had been cooled to 50 °C following autoclaving. Twenty-five mL of the solution were poured into petri dishes and left to solidify. Four µL of freshly grown *Lactobacillus* suspensions adjusted to OD_600nm_ = 1 were spotted on the agar plates and incubated for 24 h under anaerobic conditions. Subsequently, culture plates were exposed to ambient air for up to 24 h for pigment formation. Production of hydrogen peroxide was visualized by light to dark purple colonies. Each assay was performed in duplicate plates and repeated three times. Probiotic strains *L. reuteri* RC-14 and *L. rhamnosus* GG were included as positive and negative controls, respectively.

### Mercuric chloride resistance assay

For determining the strains’ resistance to HgCl_2_ (215465, Sigma-Aldrich), the *Lactobacillus* strains were grown aerobically overnight in MRS broth. The assay was performed in 96-well plates with 100 µL inocula in MRS (OD_600nm_ = 0.05) prepared from the overnight cultures and additional 100 µL of MRS broth containing HgCl_2_ dilutions to achieve end concentrations of 0.5, 0.25, 0.125, 0.0625, 0.031, 0.015, and 0.008 mmol/L. Wells containing MRS broth without HgCl_2_ were included as a control. Growth was monitored at 24 and 48 h using a BioTek Synergy 2 microplate reader for OD_600nm_ determinations. All tests were performed in triplicate wells and the experiment was repeated thrice.

### Biofilm formation

The ability of the *Lactobacillus* strains to form biofilms on plastic surfaces was quantified using the crystal violet staining method [70]. The strains were grown in MRS medium in a 6-well plate under aerobic conditions for 48 h. Subsequently, the culture medium was aspirated and adherent cells were washed twice with sterile PBS. Three mL of 0.02 % crystal violet (w/v) was added into each well to stain the surface attached bacteria and incubated for 20 min. Excess dye was rinsed off by washing the cells 5 times with distilled water. Two mL ethanol (95 %) was added to each well to redissolve the crystal violet dye from the biofilms. Following alcoholic elution, 200 µL aliquots of the eluate were transferred to a microplate and the absorbance was read at 590 nm in a Synergy 2 Multimode Microplate Reader. All tests were carried out in triplicate wells and repeated three times.

### Caco-2 cell adhesion assay

The Caco-2 cell line (HTB-37) was purchased from the American Type Culture Collection (ATCC, Rockville, MD, USA). The cells were cultured in Dulbecco’s modified Eagle’s minimal essential medium (DMEM; Life Technologies) supplemented with 10 % (v/v) heat-inactivated (30 min, 56 °C) fetal bovine serum (Life Technologies), 100 U/mL penicillin, and 100 mg/mL streptomycin (Life Technologies) at 37 °C, 5 % CO_2_in a Heracell 150i incubator (Thermo Scientific, Rockford, IL, USA). For adhesion assays, Caco-2 monolayers were prepared in 24-well standard tissue culture plates (Cellstar, Greiner Bio-One). The Caco-2 cells were seeded at a concentration of 1.0 × 10^4^ cells per well to obtain confluence and maintained for 20 days prior to the adhesion assays. The cell culture medium was replaced every other day. The number of cells per well was determined by trypsinization of the monolayer and counting using a hemocytometer. In addition, cell number and viability of the monolayer was confirmed using the PrestoBlue Cell Viability Reagent kit according to manufacturer’s instructions (Life Technologies).The adherence of *Lactobacillus* strains to Caco-2 cells was determined by the method of Fernandez et al. [71] with some modifications. Briefly, the Caco-2 monolayer was washed twice with phosphate-buffered saline (PBS) pH 7.4 (Sigma). PBS was also used to wash and adjust the lactobacilli to desired cell densities. Dilutions according to OD_600nm_ readings were used for approximation of cell densities. Viable bacterial cell numbers introduced in the adhesion assays were determined by CFU counting on MRS agar. Bacteria from an overnight culture were washed in PBS. For each adhesion assay, 500 µL of *Lactobacillus* suspension ranging from 1.5 × 10^8^ to 4.9 × 10^8^ cells per ml were added to the wells containing Caco-2 monolayers and incubated at 37 °C in 5 % CO_2_ atmosphere. After 90 min of incubation, the Caco-2 monolayers were washed three times with PBS to release non-adherent bacteria. In order to enumerate the attached viable bacteria, the mammalian cells were lysed in sterile water by repeated up and down pipetting for 10 min. Appropriate dilutions of the mixtures of lysed Caco-2 cells and bacteria were plated on MRS agar plates and incubated at 37 °C. The CFU count was determined after 48 h incubation. Data were expressed as the percent adhesion rate, i.e., the ratio between the number of adherent bacteria and the number of bacteria added to the cell monolayer. Each adhesion assay was performed in duplicate wells with cells from three successive passages (P5, P8, and P14). ATCC 33200 and LGG were included as reference strains.

### Antibiotic susceptibility testing

The antibiotic susceptibilities of selected *Lactobacillus* strains (five prairie vole isolates and two controls) were determined using a broth microdilution assay as described previously [72]. The microplate assay was adapted to MRS medium in order to support vigorous growth of lactobacilli. Eight antimicrobial drugs representing different antibiotic classes were tested: ampicillin, chloramphenicol, neomycin (A9518, C0378, N6386; Sigma-Aldrich), doxycycline, ciprofloxacin (BP2653, 449620050; Thermo Fisher Scientific), erythromycin, cephalexin monohydrate, and clindamycin HCl (E57000, C59000, C41050, Research Products International Corp.; Mount Prospect, IL, USA). Antibiotic stock solutions were prepared according to the manufacturers’ recommendations and diluted to final assay concentrations of 64–0.125 mg/L or 128–0.25 mg/L (only neomycin and cephalexin) in assay volumes of 200 μl per microplate well [72]. *Lactobacillus* inocula were adjusted to OD_600nm_ = 0.001 and plates were read after 18 h of incubation. The minimum inhibitory concentration (MIC) was defined as the lowest concentration of antibiotic giving a complete inhibition of visible bacterial growth in comparison to control wells [72]. All tests were performed twice with duplicate wells.

### Statistical analysis

All quantitative data are the average of three independent experiments ± standard deviations (mean ± SD). Statistical significance of the results was evaluated by one-way or two-way analysis of variance (ANOVA) using the IBM SPSS Statistics package (version 19) and PRISM (version 5; GraphPad Software, La Jolla, CA, USA). A *P* < 0.05 was considered statistically significant.

## Additional files

10.1186/s13099-015-0082-0 Phylogenetic tree of 16S rRNA gene sequences. The 16S rRNA gene sequences of the prairie vole isolates were obtained by Sanger sequencing of 16S amplicons (primers 8F-1491R) and next-generation genome sequencing for isolates PV012, PV021, and PV034 (NGS sequence designations). Multiple NGS contigs for these strains most likely represent distinct rDNA operons. The sequences were aligned to *Lactobacillus johnsonii* ATCC 33200 (Lj; NR_025273) and *L. rhamnosus* GG (LGG; NR_102778) 16S rDNAs. Following manual trimming of the multiple sequence alignment ends, the sequences were used to generate the depicted phylogenetic tree using the CLC Genomics Workbench Maximum Likelihood Phylogeny algorithm (UPGMA starting tree, General Time Reversible substitution model).
10.1186/s13099-015-0082-0 Antibiotic susceptibilities of the selected prairie vole *Lactobacillus* strains.
