# Supplementary figures and images for: Lactobacilli with probiotic potential in the prairie vole (Microtus ochrogaster)
Source: Gut Pathog. 2015 Dec 30;7:35. doi: 10.1186/s13099-015-0082-0 (PMC4696317; doi:10.1186/s13099-015-0082-0)

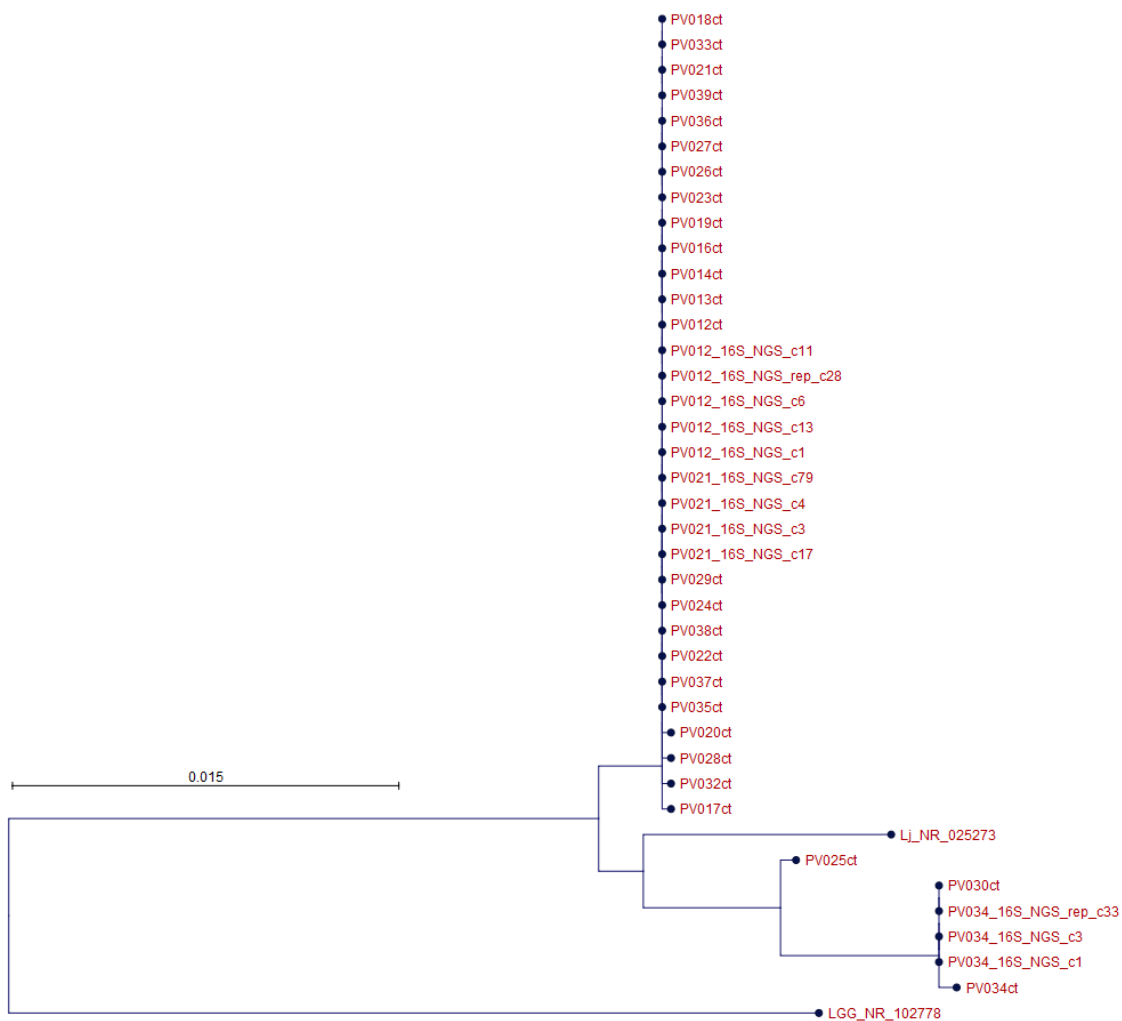

Supplement: Supplementary file 1 — 10.1186/s13099-015-0082-0 Phylogenetic tree of 16S rRNA gene sequences. The 16S rRNA gene sequences of the prairie vole isolates were obtained by Sanger sequencing of 16S amplicons (primers 8F-1491R) and next-generation genome sequencing for isolates PV012, PV021, and PV034 (NGS sequence designations). Multiple NGS contigs for these strains most likely represent distinct rDNA operons. The sequences were aligned to Lactobacillus johnsonii ATCC 33200 (Lj; NR_025273) and L. rhamnosus GG (LGG; NR_102778) 16S rDNAs. Following manual trimming of the multiple sequence alignment ends, the sequences were used to generate the depicted phylogenetic tree using the CLC Genomics Workbench Maximum Likelihood Phylogeny algorithm (UPGMA starting tree, General Time Reversible substitution model). [file 13099_2015_82_MOESM1_ESM.pdf]
