# Supplementary material for: Lactobacilli with probiotic potential in the prairie vole (Microtus ochrogaster)
Source: Gut Pathog. 2015 Dec 30;7:35. doi: 10.1186/s13099-015-0082-0 (PMC4696317; doi:10.1186/s13099-015-0082-0)
Supplement: Supplementary file 2 — 10.1186/s13099-015-0082-0 Antibiotic susceptibilities of the selected prairie vole Lactobacillus strains. [file 13099_2015_82_MOESM2_ESM.docx]

**Supplemental Table S1**

**Table S1 Antibiotic susceptibilities of the selected prairie vole *Lactobacillus* strains**

|  | Minimum Inhibitory Concentration (MIC; mg/L)^a^ of | | | | | | | |
| --- | --- | --- | --- | --- | --- | --- | --- | --- |
| **Strain** | **Ampicillin** | **Cephalexin** | **Chloramphenicol** | **Ciprofloxacin** | **Clindamycin** | **Doxycyclin** | **Erythromycin** | **Neomycin** |
| PV012 | 4 | 16 | 16 | 32 | 2 | 4 | 2 | 64 |
| PV017 | 2 | 16 | 16 | 32 | 2 | 4 | 2 | 64 |
| PV018 | 2 | 16 | 16 | 32 | 2 | 4 | 2 | 128 |
| PV019 | 2 | 16 | 16 | 32 | 2 | 4 | 2 | 128 |
| PV039 | 2 | 16 | 16 | 32 | 1 | 4 | 2 | 64 |
| Lj | 4 | 64 | 16 | 32 | 2 | 16 | 2 | >128 |
| LGG | 8 | >128 | 16 | 16 | 2 | 4 | 2 | 64 |

^a^ Data are representative for quadruplicate determinations of the MICs by broth microdilution assay in MRS Medium [[72](#_ENREF_72)].
